# Ultrasound‐assisted extraction of bioactive compounds from *Moringa oleifera* leaves for beef patties preservation: Antioxidant and inhibitory activities, half‐life, and sensory attributes

**DOI:** 10.1002/fsn3.4395

**Published:** 2024-08-09

**Authors:** A. M. S. Al‐Baidhani, Alia Z. Hashim, Haider K. Al‐Qutaifi, Asaad R. Al‐Hilphy, Muhammad Waseem, Felix Kwashie Madilo, Muhammad Faisal Manzoor

**Affiliations:** ^1^ Department of Food Science, College of Agriculture University of Basrah Basrah Iraq; ^2^ Department of Food Science & Technology, Faculty of Agriculture & Environment The Islamia University of Bahawalpur Bahawalpur Pakistan; ^3^ Food Science and Technology Department Ho Technical University Ho Ghana; ^4^ Guangdong Provincial Key Laboratory of Intelligent Food Manufacturing, School of Food Science and Engineering Foshan University Foshan China; ^5^ School of Food Science and Engineering South China University of Technology Guangzhou China

**Keywords:** antibacterial activity, antioxidant activity, beef patties, *Moringa oleifera*

## Abstract

This study aims to examine employing ultrasound‐assisted extraction of bioactive components from *Moringa oleifera* leaves and apply them in beef patties preservation, as well as antioxidant and inhibitory activities and sensory qualities. The study included studying the chemical content and minerals of the *M. oleifera* leaves, preparation of aqueous and alcoholic extracts using an ultrasound device, then exploring the extraction yield. The results proved that the extraction yield by ultrasound using ethanol at 80% was the highest, reaching 19.22%. The total phenols in the ultrasonic extract with moringa leaves aqueous extract (AMEUS) amounted to 120,755 mg/mL. Since the AMEUS exhibited the highest value of 68.308 mg/mL calic acid – eight phenolic compounds discovered by HPLC – the total content of flavonoids was also calculated. The inhibitory and antioxidant effects of moringa leaf extracts are well documented. We monitored the changes in chemical indicators, such as the value of peroxide and thiobarbituric acid, as well as the percentage of free fatty acids and physical characteristics, such as water‐carrying capacity, pH, and pigments, for storage periods 0, 4, 8, and 12 days after adding AMEUS to beef patties at a concentration of 0.5%. The patties were kept under refrigeration at 4 ± 1°C during this time. The values of peroxide number, thiobarbituric acid, free fatty acid, and metmyoglobin pigment were decreased in the beef patties treated with the AMEUS. However, they increased continuously during the cryopreservation period, and there was a significant increase in water‐holding capacity (WHC) when the beef patties were treated with AMEUS. The results also showed that adding AMEUS to beef patties improved their qualitative characteristics.

## Graphical abstract

While the total phenols in the ultrasonic extract with moringa leaves aqueous extract (AMEUS) amounted to 120,755 mg/mL, the extraction yield with ultrasound using ethanol at 80% was the highest, reaching 19.22%. The AMEUS exhibited the highest value of 68.308 mg/mL calic acid – eight phenolic compounds discovered by HPLC. While the addition of AMEUS to beef patties improved their qualitative characteristics, the leaf extracts also recorded the highest inhibitory effects on *Bacillus cereus* with the least on *Escherichia coli*.

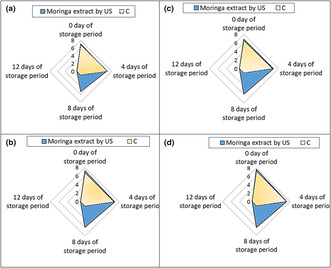

## 1 INTRODUCTION

People are looking for reasonably priced, nutritious food that does not skimp on flavor or texture because of the recent surge in population (Rakotosamimanana & De Kock, 2020). According to Aguilar et al. (2019), the food sector is always searching for new and improved ways to make food safer, better, and last longer. The nutritional value of meat is based on the calories, vital amino acids, fatty acids, vitamins, and minerals it contains, making it one of the most eaten foods. Meat reformulation nowadays often involves using natural food additives that not only make the meat last longer, but also make it healthier. One such natural preservative is extracts and powder from the fruits and leaves of the *Moringa oleifera*. Although most moringa trees are tiny or medium sized, the tallest one may reach around 10 m. It is the most beloved plant type in gardens in northwest India and has its roots in Africa and Asia. The Moringaceae family, which is a member, contains a wide range of plant types, from little herbs to enormous trees. One of the most common types of moringa plants grown is *M. oleifera*. All components of the moringa tree, including the leaves, blossoms, and seeds, are edible because of the high vitamin, mineral, and amino acid contents, as shown in several global research.

Traditional use of the leaves to treat malnutrition includes pregnant women, nursing mothers, and infants (Idris & Abubakar, 2016). *Moringa oleifera* leaves are rich in protein, vitamins A, B, and C, and minerals, including iron and calcium. When eaten raw, leaves provide a wealth of vitamins A and C. In addition to being rich in plant nutrients, they are also an excellent source of vitamin B. Compared to oranges, carrots, and other fruits, their leaves provide seven times as much vitamin C, 10 times as much vitamin A, and 17 times as much calcium. Compared to curd, milk has 9 times the protein, bananas have 15 times the potassium, and spinach has 25 times the iron. In addition to their antibacterial qualities as an antipyretic, antiepileptic, and anti‐inflammatory for the urinary tract, several plant parts – including leaves, roots, seeds, bark, fruits, flowers, and immature pods – stimulate the heart and circulatory system (Prasajak et al., 2021). Moringa leaves have a long history of being used as a medicinal food by Asian populations. International research has shown that these leaves are an excellent dietary supplement due to their high concentration of phytochemicals, vitamins, minerals, vital amino acids, and proteins (Islam et al., 2021; Syeda & Riazunnisa, 2020). Carotenoids, polyphenols, phenolic acids, flavonoids, alkaloids, glucosinolates, isothionitrates, tannins, and saponins are physiologically active substances in the leaves; because of their high nutritional content and abundance of antioxidants, it has medical uses and has helped alleviate symptoms of diabetes, high blood pressure, malaria, and typhoid fever. In addition to regulating thyroid hormone levels, it enhances liver and kidney function and protects against stomach ulcers. Infections can be a side effect of cirrhosis, liver damage, cancer, and excessive cholesterol (Islam et al., 2021; Vergara‐Jimenez et al., 2017).

This research aimed to compare the antioxidation activities of alcoholic and water‐based extracts with and without ultrasonication to identify the superior extract. Also, to make the beef patties last longer in the fridge, they used the finest extract. I will also look at how it changes the chemical properties of beef patties.

## 2 MATERIALS AND METHODS

According to the method described by Tlay et al. (2024), the chemical content of moringa leaves includes moisture, and protein, fat, and ash contents, according to AOAC (2007). Minerals were also estimated using the method mentioned in Aidos (2002).

### 2.1 Preparation of extracts

#### 2.1.1 Alcoholic extracts

I used 100 g of dried leaves and made the alcoholic extracts following the procedure of Elmastas et al. (2015) with a few tweaks. In a 5:1 weight/volume extraction ratio, we used 100% ethyl and methyl alcohol. After thoroughly mixing the mixture, we placed the samples in a vertical shaker set at 30°C for 24 h. Whatman No. 1 filter paper was used to filter the extract, and a rotary vacuum evaporator was used to concentrate the filtrate. We let the filtrate evaporate the solvent at room temperature in the laboratory. Until needed, the extracts were refrigerated in opaque vials with tight lids.

#### 2.1.2 Ultrasonic extraction

A combination of ethanol and water was used in a ratio of 4:1 by volume/volume for the extraction process. Then, following a slightly modified version of the method described by Shepelev et al. (2016), 100 mL of the alcoholic mixture (ethanol and methanol) was combined with water, and the water extracts were mixed with 10 g of dry leaf powder to make 100% aqueous extracts. The samples were heated to 30°C and shaken vertically for 24 h. After being heated to 33°C for 15 min at a frequency of 20 kHz, it was subjected to an ultrasonic apparatus. The next step was to filter it using Whatman No.1 filter paper after centrifuging it for 15 min at 3500 rpm. There were three rounds of precipitate re‐extraction. After drying it at laboratory temperature, we weighed the filtrate and kept it in opaque, sealed containers in the fridge until we needed it.

### 2.2 Determination of total phenols

Shepelev et al. (2016) estimated the total phenol content. We measured the absorbance at a wavelength of 725 nm. Using a reference solution of gallic acid ranging from 0 to 500 mg/mL, the amount of phenols in the extracts was determined by graphing the relationship between acid concentration and absorbance at a wavelength of 725 nm (Figure S1).

### 2.3 Determination of total flavonoids

Khan et al. (2012) proposed a methodology for estimating total flavonoid content. We measured the absorbance at 510 nm. Using a graphical link between compound concentration and absorption, the quantity of flavonoids was determined from a standard solution of Rutin compound with concentrations ranging from 0 to 150 mg/mL (Figure S2).

### 2.4 HPLC analysis of bioactive compounds of *M. oleifera* leaves

The study followed the same high‐performance liquid chromatography (HPLC) procedure described in Ngamsuk et al. (2019). The researchers could determine the percentages of eight compounds using a SYKAMN HPLC system (Germany) and a C18‐ODS column (250 × 4.6 mm, 5 μm). There was an injection of 100 μL of samples into the system. Mixing 1 mL/min of solvent A (95% acetonitrile and 0.01% trifluoroacetic acid) and solvent B (5% acetonitrile and 0.01% trifluoroacetic acid) made up the mobile phase. The gradient program follows: beginning at 0 min, followed by 25% A for 5–7 min, 40% A for 7–13 min, and finally, returning to the starting circumstances. A 278‐nm UV–visible detector was used for phenolic compound detection.

### 2.5 Measurement of antioxidant activity

Osawa and Namiki's (1981) approach was used to test the antioxidant activity of alcoholic and water‐based extracts using ferric thiocyanate. We tested the absorbance at 500 nm. Using the following equation, we were able to determine the linoleic acid peroxide inhibition %:

(1) $$\text{Antioxidant activity}\%=100-\left[\frac{\text{Absorbance of sample}}{\text{Absorbance of the control}}\right]\times 100$$

### 2.6 Reducing power

We used the procedure outlined in El Jemli et al. (2016) to find the plant extracts' reduction power. After incubating a combination of 1 mL of 10% potassium ferricyanide solution, 1 mL of 0.2 M phosphate regulator solution with a pH of 6.6, and 1 mL of 5 mg/mL extracts at 50°C for 20 min, 1 mL of 1% Trichloroacetic acid added. The combination underwent a 10‐min centrifugation run at 3500 rpm. After removing 1 mL of the solution's top layer, add 1 mL of distilled water and 2 mL of ferric chloride (0.1%). After that, a wavelength of 700 nm was used to measure the absorbance. Components in the reaction mixture with an increased absorbance have a strong reducing power.

### 2.7 Chelating of ferrous ion

Following the methodology outlined by Sellal et al. (2019), the ferrous ion‐binding capabilities of the extracts were evaluated. As reference solutions, we utilized EDTA and citric acid. Following this equation, we were able to determine the extracts' ferrous ion binding capabilities:

(2) $$\text{Chelating}\;\left(\%\right)=\left[\frac{\text{Absorbance of the control}-\text{Absorbance of sample}}{\text{Absorbance of the control}}\right]\times 100$$

### 2.8 Scavenging hydrogen peroxide

Zhang (2000), as described in Sharma and Singh (2012), used a method to estimate the extracts' hydrogen peroxide (H_2_O_2_)‐capturing capabilities. They took 0.1 mL of hydrogen peroxide (at a concentration of 5 mg/mL, 0.1 mM) and 0.1 mL of the extracts and added two drops of ammonium molybdate solution to the mixture. After adding 10 mL of 2 M sulfuric acid (H_2_SO_4_) and 7 mL (3%) of 1.8 molar potassium iodide (KI) was added to the mixture. Sodium thiosulfate (NaS_2_O_3_) at a concentration of 5.09 mmol was used to level the reaction mixture until the yellow color disappeared. The trapping ability was then determined using the following equation:

(3) $$\text{Scavenging}\;\left(\%\right)=\left[\frac{V_{2}-V_{1}}{V_{1}}\right]\times 100$$

V_1_ = the volume of NaS_2_O_3_ for the control, V_2_ = the volume of NaS_2_O_3_ for the sample.

### 2.9 Bacteria of strains

The isolated bacteria were used as an antibacterial effect in the study of *Escherichia coli*, *Staphylococcus aureus*, *Bacillus cereus*, and *Pseudomonas aeruginosa*; they were obtained from the Department of Food Science, Agriculture College Basrah, Iraq.

### 2.10 Antibacterial activity of *Moringa* leaves extract

Following the protocol outlined by Albuquerque et al. (2006), the antibacterial activity of a 0.5% concentration of an ultrasonic aqueous extract of *M. oleifera* leaves was assessed on Mueller–Hinton agar media. The diameter of the zone of inhibition was measured by spreading 25 μL of a bacterial suspension diluted to 10^8^ CFU/mL onto plates. The wells (6 mm) were filled with approximately 100 μL of *M. oleifera* extract at various concentrations and then allowed to diffuse at room temperature for 2 h. As a control, we utilized the distilled water as a positive control to make the extract, and we incubated bacterial plates at 37°C for 24 h. Measuring the inhibition zones against the examined microorganisms allowed us to determine the antibacterial activity.

### 2.11 Preparation of beef patties

We used a machine with holes 3 mm in diameter to mince 700 g of beef from the thigh region. We utilized two treatments: A control sample (C) containing no extracts was the first treatment. The second treatment involved adding a 0.5% concentration of an aqueous moringa extract by ultrasonic (AMEUS) to the meat. We inflated plastic bags with air and put the beef patties inside. To separate the patties, we used wax paper and kept them in the fridge at 4°C for 0–12 days. We measured them every 4 days.

### 2.12 Chemical indicators

#### 2.12.1 Peroxide value (PV)

Miadonye et al. (2023) provided the peroxide value methodology. Transferring 3 g of beef patties from the middle of the sample (not the surface) to a 250‐mL conical flask, we added 50 mL of the solvent mixture of glacial acetic acid and isooctane (3:2), then 1 mL of saturated potassium iodide solution. We allowed the mixture to react for 60 s while stirring it by hand. After that, pour 100 mL of distilled water into it and shake it well. Finally, prepare the plank as before by adding 1 mL of starch solution, titrating with 0.1 sodium thiosulfate solution, and shaking the mixture until the blue color fades.

(4) $$\mathrm{PV}=\frac{1000\left(V1-V0\right)c\;}{m\;}$$

PV is peroxide value – mEq/kg.

V1 is the volume of sodium thiosulfate solution consumed in the titration of involving meat patties (cc), V0 is the volume of sodium thiosulfate consumed in the blank titration that does not contain sample (cc), *c* is the concentration of sodium thiosulfate solution (0.1 N), and *m* is the mass of meat patties analyzed.

#### 2.12.2 Thiobarbituric acid

To determine the worth of thiobarbituric acid (TBA), Soltanizadeh and Ghiasi‐Esfahani (2015) employed the technique. Mixing 50 mL of 20% trichloroacetic acid (TCA) with 20 g of beef patties took 2 min. We blended the contents of the blender with 50 cc of water, washed it, and filtered it through a Whatman No. 1 filter paper. Finally, 5 mL of 0.01 M TBA was added to 5 mL of TCA extract and left at 100°C for 1 h. Our UV–vis spectrophotometer assessed the pink color solution's absorbance at 532 nm. Malonaldehyde milligrams per kilogram of sample was the unit of measurement for total bromide content.

#### 2.12.3 Free fatty acids

Al‐Baidhani and Al‐Mossawi (2019a) cited a technique that determines the acid number by first determining the acid value and then using that proportion to determine the amount of free fatty acid (FFA):

(5) $$\text{Acid value}=\left[\frac{\text{milliliters of potassium hydroxide}\times 5.61}{\text{weight of sample}\;}\right]$$

(6) $$\mathrm{FFA}\;\left(\%\right)=\left[\frac{\text{Acid value}\;}{2}\right]$$

### 2.13 Kinetic reaction of PV development during storage and half‐life

We used a kinetic model to account for the rise in peroxide value (PV) seen during storage to determine the half‐life. We used the zero and first kinetics models, as mentioned in Equations 7 and 8, to compute the increase in PV (meq/kg oil) over the storage period (Sapei & Hwa, 2014):

(7) $$\mathrm{PV}={\mathrm{PV}}_{0}-k_{0}t$$

(8) $$\mathrm{PV}={\mathrm{PV}}_{0}\;\exp.\left(-k_{1}t\right)$$

Because the process is increasing PV during storage, the positive sign was used as illustrated in Equations 8 and 9:

(9) $$\mathrm{PV}={\mathrm{PV}}_{0}+k_{0}t$$

(10) $$\mathrm{PV}={\mathrm{PV}}_{0}\;\exp.\left(k_{1}t\right)$$

where PV and ${\mathrm{PV}}_{0}$ are the peroxide value (meq/kg oil) at any given time (*t* (day)) and at zero time, respectively, $k_{0}$ and $k_{1}$ are the constant rates of the zero kinetic model (meq/kg oil) and the first kinetic model (1/day), respectively. The constants ($k_{0}$ and $k_{1}$) were found by using Solver in the Excel program in 2013. The half‐life (*t*_1/2_) to increase PV in chicken meat is given in the Equation 11:

(11) $$t_{1/2}=\frac{-\ln\left(0.5\right)}{k}$$

$k=k_{0}$ is the constant rate for the zero‐order model, $\;k=k_{1}$ is the constant rate for the first‐order model.

### 2.14 Physical properties

#### 2.14.1 Water‐holding capacity

Al‐Tai and Al‐Mossawi (1992) described a technique for estimating the water‐carrying capacity of prepared samples by mixing 10 g of meat with 20 mL of distilled water. There was a good mixing of the materials. After that, everything was moved to a graduated cylinder with a funnel and filter paper attached. Thirty minutes later, the volume was measured after the spray, and the data were recorded accordingly. The following is the formula for determining the water‐carrying capacity:

Water‐holding capacity (WHC)=Total water amount (mL)−Amount of water in the inserted cylinder (mL)

#### 2.14.2 pH value

Al‐Tai and Al‐Mossawi (1992) cited Mendenhall (1989) as the technique to estimate the pH value. For this experiment, we combined 10 g of sample with 20 mL of distilled water, let it sit for 5 min, and then measured the pH.

#### 2.14.3 Pigments

Following the procedure described by Lee et al. (1998), we employed a phosphate buffer with a concentration of 0.04 molarity and a pH of 6.8 to determine metmyoglobin staining. We used a spectrophotometer to measure the absorbance at three different wavelengths: 700, 572, and 525 nm. We used the following equation to get the metmyoglobin pigment percentage:

(12) $$\text{Metamyoglobin pigment}\;\left(\mathrm{Met}-\mathrm{Mb}\right)\left(\%\right)=1.39-\frac{A_{572}–A_{700}}{A_{525}–A_{700}}\times 100$$

Using a spectrophotometer and the method described by Broumand et al. (1958), the concentrations of myoglobin and oxymyoglobin in ground beef discs were measured. The ratio of absorption intensities at 597 and 473 nm was used to estimate myoglobin, and the percentage of oxymyoglobin was determined using the following equation:

(13) $$\text{Oxymyoglobin}\;\left(\%\right)=100–\left(\text{Metamyoglobin}\;\left[\%\right]+\text{Myoglobin}\;\left[\%\right]\right)$$

#### 2.14.4 Cooking yield

Khomola et al. (2021) reported that ultrasonography was used to measure the cooking output of beef patties treated with aqueous extract. To find the cooking yield, we divided the weight of the cooked product by its raw, uncooked weight and then multiplied the result by 100.

#### 2.14.5 Cooking shrinkage

According to Kenawi and Abd El‐Hameed (2018), the cooking shrinkage of cooked meat patties was determined. The shrinkage value of cooked beef patties was determined as follows:

(14) $$\text{Shrinkage value}\;\left(\%\right)=\frac{\left(R.T-C.T\right)+\left(R.D-C.D\right)}{\left(R.T+R.D\right)}\times 100$$

where R.T = raw sample thickness, C.T = cooking sample thickness, R.D = raw sample diameter, C.D = cooked sample diameter.

#### 2.14.6 Color measurement

Color compounds were measured for beef patties supplemented with moringa leaf extract as whiteness (L*), redness (a*), and yellowness (b*) by color grab software with a smartphone device, according to Peng et al. (2019). At three locations, determine the average values of L*, a*, and b* during the measurements.

### 2.15 Sensory evaluation

A sensory evaluation was carried out, which included flavor, tenderness, juiciness, and general acceptance of the meat patties treated with moringa extract to determine consumer acceptance according to the methods described in Kenawi and Abd El‐Hameed (2018), where several experts participated in this evaluation, and according to the numerical assessment ranging from 1 (very bad) to 10 (excellent) that was used for assessment.

### 2.16 Statistical analysis

The data were analyzed using the SPSS version 21 at the 0.05 level. The least significant difference (LSD) was employed to compare the means of the treatments. The current study's experiments were all done in triplicate.

## 3 RESULTS AND DISCUSSION

### 3.1 Chemical content

The chemical composition of the moringa leaf powder is shown in Table S1. The moisture, protein, fat, and ash percentages were 4.98%, 27.37%, 5.11%, and 6.88%, respectively. The results demonstrated that the moringa leaf powder has a dietary fiber percentage of 19.27% and a carbohydrate percentage of 36.39%, which is an outstanding combination. El‐Massry et al. (2013) examined the chemical composition of powdered dry moringa leaves and found that it had 5.48% water, 26.79% protein, 4.98% fat, 7.92% fiber, 18.67% carbohydrates, and 35.90% ash. In their investigation of the chemical makeup of moringa leaf powder, Shokry (2017) and Peñalver et al. (2022) came to similar conclusions.

In Table S1, we can see that the mineral elements calcium, phosphorus, iron, magnesium, potassium, and zinc all had concentrations of 2031.55, 3390.77, 26.87, 7.49, 389,055, 1422.66, and 3.22 mg/100 g, respectively. Amabye and Gebrehiwot (2015) also found mineral element levels that were consistent with these findings: calcium (Ca), phosphorus (P), iron (Fe), sodium (Na), magnesium (Mg), potassium (K), and zinc (Zn): 2016.5, 1845, 19.37, 8.13, 322.5, 1845, and 1.0 mg/100 g, respectively. Consistent with what Debebe and Eyobel (2017) found, the outcomes were as expected. When comparing the mineral composition of moringa leaves from various soil types.

### 3.2 Extract yield

With the use of the ultrasonic extraction equipment and various solvents, we were able to get a somewhat variable extraction yield. Table 1 shows that there was a significant difference (*p* ≤ .05) in the percentages of the extraction yield when using different solvents, specifically ethanol 80% (Eu), methanol 80% (Mu), and an ultrasonic device using water 100% (AMEUS: aqueous moringa extract by ultrasonic), with respective percentages of 12.33%, 15.30%, 8.13%, 19.22%, and 11.41%.

**TABLE 1:** Extract yield, total phenols, and total flavonoids are affected by the extraction method.

Extraction method	Extract yield (%)	Total phenols (mg/mL)	Total flavonoids (mg/mL)
AMEUS	12.33 ± 1.34^c^	120.75 ± 3.99^a^	68.30 ± 2.01^a^
E	15.3 ± 1.08^b^	94.37 ± 2.67^b^	18.24 ± 1.09^d^
M	8.13 ± 1.10^e^	80.711 ± 2.11^c^	30.51 ± 0.98^b^
Eu	19.22 ± 2.09^a^	70.82 ± 1.34^d^	13.71 ± 0.88^e^
Mu	11.41 ± 0.98^d^	60.48 ± 1.12^e^	19.22 ± 0.97^c^

*Note*: The different superscript alphabets refer to significant differences among treatments at a significant level of .05.

Abbreviations: AMEUS, aqueous moringa extract by ultrasonic; E, ethanol; Eu, ethanol and ultrasound; M, methanol; Mu, methanol and ultrasound.

The two most common plant byproducts, phenols and flavonoids, have unique biological functions. To avoid cardiovascular disease, aging, and oxygen scavenging, phenolic compounds – compounds with natural antioxidant activity – are needed. These compounds are highly effective in stabilizing free radicals – radicals that are not bound (Sahumena et al., 2021). Table 1 showed that the total phenols concentration in extracts from moringa leaves differed significantly (*p* < .05). According to the data, the aqueous extracts from (Wu) had the most significant concentration at 120,755 mg/mL, followed by (E) at 94.37 mg/mL and (M) at 80 mg/mL. The concentrations of (Eu) and (Mu) were 70.822 and 60.488 mg/mL, respectively. Ultrasound waves can enhance the extraction process by creating cavitations – transient cavities that form and collapse in a fluid. Cavitations like this help disrupt the cellular structure of plant materials, making it easier to extract bioactive compounds. The findings agreed with Pari et al. (2007) and Singh et al. (2009), as the aqueous extracts demonstrated 105.4 and 118 mg/mL concentrations, respectively. Although the total phenol concentration decreased and reached 20.5 mg/mL in the aqueous extract of the leaves, Chumark et al. (2008) noted that the variable extraction processes and procedures and the different plant culture conditions are to blame for these outcomes. Some phenolic compounds are in free form and some are linked. The total phenols may vary depending on the extraction process and solvents utilized. Results corroborated those of Zullaikah et al. (2019) for phenolic component extraction using ethanol; the total phenol content reached 87.11 mg/mL.

### 3.3 Total content of flavonoids

Flavonoids possess diverse chemical and biological characteristics; they are a class of plant metabolic byproducts. Flavonoids have several beneficial effects, including antioxidant, anticancer, antidiabetic, and antiatherosclerotic. Based on the component rutin, the total flavonoid concentration in moringa leaf powder extracts differed significantly (*p* < .05), as shown in Table 1. According to the results, different solvents and extraction methods yielded different amounts of flavonoids. The aqueous ultrasonic extracts (AMEUS) had the greatest rutin concentration at 68.308 mg/mL, followed by the equivalent units (Eu) at 13.717 mg/mL, and the respective amounts of molecular weight 30,509, 19,221, and 18,245 mg/mL. Since the alcoholic extracts produced a value ranging from 15 to 27 mg/g, the results agreed with those of Sreelatha and Padma (2009), who studied the total flavonoid concentration. This variation in flavonoid concentration may be because the extraction solvents were not as effective as one another or because the flavonoids were not soluble in the same solvents.

### 3.4 Identification of phenolic compounds in moringa leaf extracts

Using ultraviolet light at 278 nm, the major total phenolic components in moringa leaf extracts were detected using HPLC. See Figures S1 and S2 for chromatograms of the eight reference compounds utilized for comparison: quercetin, caffeic acid, rutin, kaempferol, apigenin, ferulic acid, chlorogenic acid, and gallic acid. According to their appearance by HPLC technology, the phenolic compounds of moringa leaf extract were as follows: retention time, percentage, and concentrations in ppm of gallic acid (70.11 ppm), rutin (62.15%), apigenin (42.88%), and caffeic acid (42.15%). Next, we have the chemicals with concentrations of 33.65 ppm for quercetin, 30.47 ppm for ferulic acid, 26.49 ppm for apigenin, and 16.44 ppm for chlorogenic acid. By Abo El‐Fadl et al. (2020) and Abdallah et al. (2023), moringa leaf extracts contained the same compounds (quercetin, caffeic acid, rutin, kaempferol, apigenin, ferulic acid, chlorogenic acid, and gallic acid) in varying amounts. This variation could be because of differences in the plant, soil, climate, genetics, and dietary factors.

### 3.5 Antioxidant efficacy

Several pathways by which plant extracts high in phenolic compounds exhibit antioxidant action. As antioxidants, different compounds work in different ways. Some chelate metals that catalyze oxidation, while others donate hydrogen and electrons. Still, others scavenge free radicals. Due to its intricacy, the evaluation of natural extracts' antioxidant activity involves the execution of many tests. So, to find out how effective they may be as natural antioxidants, scientists have conducted a battery of antioxidant tests. Table 2 shows substantial differences (*p* < .05) in antioxidant activity among moringa leaf powder extracts. In terms of antioxidant activity, the results demonstrated that the aqueous extracts (AMEUS) were very effective at 83.57%, the extract (Eu) at 81.87%, the mixture (Mu) at 80.91%, and the solids (E) and liquids (M) at 79.22% and 78.88%, respectively. Using ultrasound to aid extraction has been linked to higher antioxidant activity in moringa leaf extracts, which raises the possibility that more bioactive extracts might be produced this way (Dadi et al., 2019).

**TABLE 2:** Antioxidant efficacy of *Moringa* leaf extracts.

	Antioxidant activity %	Reducing power %	Chelating ability Fe^2+^	Scavenging ability H_2_O_2_ %
AMEUS	83.57 ± 0.83^a^	1.465 ± 0.77^a^	82.77 ± 0.88^a^	82.87 ± 0.86^a^
100% Ethanol	79.22 ± 0.72^c^	1.249 ± 0.80^d^	78.76 ± 0.73^d^	78.44 ± 0.85^d^
100% Methanol	78.88 ± 0.91^d^	1.155 ± 0.81^e^	77.91 ± 0.91^e^	76.81 ± 0.80^e^
US + 80% Ethanol	81.87 ± 0.89^b^	1.345 ± 0.86^b^	80.23 ± 0.80^b^	81.71 ± 1.04^b^
US + 80% Methanol	80.91 ± 0.76^b^	1.295 ± 0.61^c^	79.66 ± 0.96^c^	80.56 ± 0.72^c^

*Note*: The different superscript alphabets refer to significant differences among treatments at a significant level of .05.

Abbreviation: AMEUS: aqueous moringa extract by ultrasonic.

By lowering the Fe^3+^/ferric cyanide complex to Fe^2+^, the reducing force serves as a potent measure of antioxidant activity; this, in turn, proves that the extracts contain antioxidants, which reduce Fe^3+^ to Fe^2+^. Active compounds with a high reducing power (high absorbance reading) can donate an electron more effectively when they form a blue or green dye at a wavelength of 700 nm. This allows them to interact directly with free radicals, stabilizing them and ending their reproduction process.

Table 2 shows a substantial difference (*p* < .05) in reduction power among extracts, with variations dependent on solvent type and extraction procedure. Results showed that the investigated extracts (AMEUS), (M), (E), (M), (Eu), and (Mu) had successive absorbance values of 1.465, 1.249, 1.155, 1.345, and 1.295. Plant extracts may contain active compounds that bind ferrous ions and inhibit oxidation. These compounds, ascorbic acid, carnosine, and certain acids, have a chelating property that binds ions of metallic elements that stimulate oxidation. This suppresses oxidation by preventing free radical production. Amino acids, peptides, and proteins bind metal in extracts with phenolic and nonphenolic substances like ascorbic acid. Tripartite metals, such as ferric complexes with anthocyanin color, are simpler to deal with than ferrous double‐charged iron (Geetha et al., 2011). Moringa leaf extracts bound ferrous ions well. Table 2 shows that AMEUS had the highest binding capacity (82.77%) compared to extracts (Eu) and (Mu) (80.23% and 79.66%, respectively), with a significant difference (*p* < .05). Extracts (E) and (M) were 78.76% and 77.91% susceptible.

Upon interaction with iron and copper ions, hydrogen peroxide can create hydroperoxide radicals, the buildup of which might lead to harmful consequences. The presence of active chemicals inhibits the generation of hydrogen peroxide radicals, which means that they play a crucial role in regulating cellular H_2_O_2_ levels, which is significant for biological reasons. According to Thampi and Jeyadoss (2015), there is a direct correlation between this and the existence of substances that might prevent the production of H_2_O_2_. Researchers found that extracts made from powdered moringa leaves might absorb hydrogen peroxide. For (W), the results were 82.87%, 81.71%, 80.56%, 78.44%, and 76.81%; for (Eu), (Mu), (E), and (M), the results were 76.81%, indicating substantial variation (*p* < .05) in the extracts' capacity to capture.

Consistent with the earlier results, Singh et al. (2009) found that moringa extracts had an antioxidant activity of 85.77%. Extracts have high reducing power, peroxide capture, and ferrous ion‐binding properties. Moringa plant leaves contain potent antioxidants and have high reducing power, ferrous ion‐binding capacity, and peroxide scavenging ability, according to Sahumena et al. (2021), Chumark et al. (2008), Iqbal and Bhanger (2006), and others. Moringa leaves contain several antioxidants that may benefit human health. These compounds have been used as food additives in the nutraceutical, functional food, pharmaceutical, and biotechnological sectors.

### 3.6 Antibacterial activity

Table S3 displays the antibacterial activity of ultrasonic extract with moringa leaves aqueous extract (AMEUS) at a concentration of 0.5%. The results demonstrated that the microorganisms under study were sensitive to different levels of extraction based on the inhibition zone; the AMEUS inhibitory zone ranged from 7 to 11 mm, with 0.05% showing the most potent antibacterial action against *B. cereus* (11 mm zone of inhibition) and a weak effect against *E. coli* (7.5 mm zone of inhibition). Al_husnan and Alkahtani (2016) found that an aqueous moringa leaf extract strongly inhibited *B. cereus* but weakly inhibited *P. aeruginosa*, *S. aureus*, and *E. coli*. Khamael Ali Kareem et al. (2020) found that various extraction series had varying sensitivities on the inhibitory zone. Extracts from *M. oleifera* had a zone of inhibition ranging from 9–17 mm at 10 mg/L to 7–13 mm and 10–12 mm at 20 and 30 mg/L, respectively, against bacteria.

### 3.7 Chemical indicators

In minced beef patties held in cold storage ±4 m, peroxide, thiobarbituric acid, and FFAs rose continually, and the findings in Table 3 show a vital variation (*p* ≤ .05) in this regard. However, this improvement was minor when compared with the C growth. During the same storage periods, the peroxide levels in the C sample were 0.049, 3.19, 4.22, and 5.47 mEq/kg, but in the treated patties by AMEUS, they were 0.048, 0.96, 1.69, and 2.31 mEq/kg, respectively. The creation of peroxides, which happens when lipids oxidize during storage, causes the levels to rise. However, treatment with extracts lowers the formation of peroxides because they include chemicals that hinder their synthesis.

**TABLE 3:** Chemical indicators of beef patties.

Chemical indicators	Treatments	Storage period (day)			
		0	4	8	12
PV (mEq/kg)	AMEUS	0.048 ± 0.01^a^	0.96 ± 0.92^b^	1.69 ± 0.81^c^	2.31 ± 0.89^d^
	C	0.049 ± 0.01^a^	3.19 ± 0.77^d^	4.22 ± 0.80^e^	5.47 ± 0.81^e^
TBA (mg malonaldehyde/kg)	AMEUS	0.44 ± 0.08^A^	0.48 ± 0.13^A^	0.79 ± 0.74^B^	1.12 ± 0.91^C^
	C	0.46 ± 0.08^A^	0.78 ± 0.24^B^	1.89 ± 0.77^D^	3.86 ± 1.24^E^
FFA (%)	AMEUS	0.23 ± 0.16^a^	0.26 ± 0.08^a^	0.48 ± 0.08^b^	0.69 ± 0.16^c^
	C	0.25 ± 0.17^a^	0.29 ± 0.16^a^	1.71 ± 1.69^d^	2.31 ± 0.81^e^

*Note*: The different superscript alphabets refer to significant differences among treatments at a significant level of .05.

Abbreviations: AMEUS, ultrasound extraction; C, control; FFA, free fatty acid; PV, peroxide value; TBA, thiobarbituric acid.

A variation (*p* < .05) was seen between AMEUS and C regarding the TBA value, as indicated in Table 4. Increasing the storage duration led to an increase in TBA. Compared to the C sample, which showed a growth of 0.46, 0.78, 1.89, and 3.86 mg malonaldehyde/kg for the exact storage durations, this one showed a minor increase, reaching 0.44, 0.48, 0.79, and 1.12 mg malonaldehyde/kg for 0, 4, 8, and 12 days of storage, respectively. Lipase and lipolytic bacteria create FFAs from lipids. The results demonstrated that the C sample significantly increased FFA percentages more than the AMEUS sample. This was because the C sample had values of 0.25%, 0.29%, 0.69%, and 2.31% for storage periods of 0, 4, 8, and 12 days, respectively, whereas the AMEUS sample had percentages of 0.23%, 0.26%, 0.48%, and 0.69%.

**TABLE 4:** Physical properties of patties treated by US and C during the storage period.

Physical properties	Treatments	Storage period (day)			
		0	4	8	12
WHC (mL)	AMEUS	18.20 ± 0.72^a^	15.11 ± 0.93^e^	16.38 ± 0.81^c^	15.22 ± 1.2^d^
	C	17.11 ± 0.47^b^	15.11 ± 1.96^e^	13.24 ± 0.91^f^	11.88 ± 0.81^g^
pH	AMEUS	5.61 ± 1.24^D^	5.69 ± 0.94^D^	5.93 ± 1.41^C^	6.36 ± 0.178^B^
	C	5.68 ± 0.94^D^	5.99 ± 0.81^C^	6.48 ± 0.94^B^	7.11 ± 0.34^A^
Cooking yield (%)	AMEUS	70.57 ± 2.35^a^	67.11 ± 0.94^b^	64.52 ± 4.71^c^	63.37 ± 3.2^d^
	C	60.68 ± 1.6^e^	60.22 ± 2.30^e^	59.95 ± 0.47^e^	59.11 ± 0.81^f^
Cooking shrinkage (%)	AMEUS	35.80 ± 0.3^g^	37.29 ± 1.88^f^	38.62 ± 0.81^e^	39.36 ± 0.81^d^
	C	39.11 ± 0.81^d^	40.23 ± 1.69^c^	41.52 ± 0.45^b^	43.77 ± 1.4^a^
L*	AMEUS	33.52 ± 0.77^a^	32.41 ± 0.86^b^	31.87 ± 0.91^d^	30.41 ± 0.77^e^
	C	32.81 ± 0.81^b^	30.55 ± 0.68^c^	30.11 ± 0.87^e^	28.89 ± 0.76^f^
a*	AMEUS	17.81 ± 0.77^B^	14.46 ± 0.87^D^	14.22 ± 0.65^D^	14.11 ± 0.87^D^
	C	15.77 ± 0.81^C^	13.69 ± 0.62^E^	13.47 ± 0.63^E^	38.11 ± 0.74^A^
b*	AMEUS	15.91 ± 0.83^a^	15.23 ± 0.87^bc^	15.11 ± 0.66^c^	14.31 ± 0.74^d^
	C	15.52 ± 0.65^b^	13.57 ± 0.96^e^	13.34 ± 0.79^e^	11.41 ± 0.64^f^

*Note*: The different letters refer to significant differences among treatments for every property at a level of .05.

Abbreviations: C, control; US, ultrasound extraction.

In addition to their antibacterial and antioxidant properties, the data demonstrate that the extracts aid in extending the product's shelf life by preventing secondary lipid oxidation. The antioxidant characteristics of moringa leaves provide some relief in this regard. As an example, a study conducted by Rahman et al. (2020) indicated that the addition of moringa leaf extracts to goat meat reduced the rise in chemical markers (POV, TBA, and FFA) when compared to a control sample that did not include extracts and did not undergo storage. Confirming prior studies on plant‐extract beef patties (Ibrahim et al., 2018; Rahman et al., 2020), the results are satisfactory. The results obtained by Al‐Baidhani and Al‐Mossawi (2019b) agree that the values of POV, TBA, and FFA increase as the storage duration increases.

### 3.8 Mathematical modeling of PV development during storage and half‐life

The results in Table S4 illustrated that the rate constant (*k*_o_) of treated meat by AMEUS was 0.19610 using the zero‐order model, which gave a higher *t*_1/2_ (3.53453 days). Therefore, the zero‐order model can predict PV development during storage because the RMSE was lower (0.086541) than C (0.644545). Also, the determination coefficient (*R* ^2^) using AMEUS was higher (0.996202) than C (0.963161). First‐order model cannot be used to predict PV during storage because the statistic parameters related to RMSE were higher, and *R* ^2^ was lower than the zero‐order model for AMEUS and C. Generally, the *t*_1/2_ for meat treated by AMEUS was higher than the C by 152.64%.

### 3.9 Physical and cooking properties

According to Table 4, the water holding capacity (WHC) of beef patties treated with AMEUS and C were significantly different (*p* < .05). Compared to C, the results showed that AMEUS added to beef patties decreased the proportion of water loss. For example, after 12 days of cold storage with AMEUS, the WHC dropped from 18.2 to 15.22 mL, but after 12 days of storage at C, it decreased from 17.11 to 11.88 mL. This decline was slow since the WHC values were 17.11 and 16.38 mL for the AMEUS samples over the 4‐ and 8‐day storage periods, respectively, and 15.11 and 13.24 mL for the C samples for the same two storage periods. One possible explanation for the high water‐carrying capacity of meat samples treated with AMEUS is the presence of phenolic compounds. These compounds are antioxidants, protecting fats from oxidation induced free radicals and preserving the cell membranes surrounding muscle fibers. This, in turn, enhances the meat's ability to withstand water absorption. Meat helped to retain water during storage, and these extracts contributed to providing stability to the cellular structure of meat and protecting the components of sarcoplasm and fluids in the membranes during meat storage from oxidative damage, which results in less loss of exudative fluid upon defrost and a reflection of this in the susceptibility to loss during cooking and an improvement increase in the meat's capacity to hold water during storage because the safety and protection of these membranes, as well as limiting their rupture, help to preserve the meat's cellular components. Additionally, the high pH encourages or increases water binding to the protein inside muscle cells, which results in water penetration from the outside of the cells to the inside. Finally, the increase in water‐carrying capacity is due to the increased ability of meat proteins to absorb water because they are less soluble during storage (Al‐Baidhani et al., 2024; Mashau et al., 2021).

### 3.10 Color properties

Table 4 shows that the color values of the beef patties supplemented with moringa leaf extract changed as a function of refrigeration time. Specifically, the lightness values (L*) of the AMEUS‐treated sample rose from day 0 to 12 days compared to the C‐treated sample, where the L* values dropped. For samples treated with AMEUS at 0, 4, 8, and 12 days of storage, the corresponding L* values were 33.52, 32.41, 31.87, and 30.41, respectively. As for the Cs, respective L* values were 32.81, 30.55, 30.11, and 28.89. High meat and muscle pigment redness may explain C's low L* value. Natural antioxidants in AMEUS may explain why it treats more burgers. Patties treated with AMEUS significantly increased (*p* < .05) in a* values. The C sample had lower a* values during storage. Low C a* levels during storage imply myoglobin oxidation, generation, and lipid oxidation in beef products. Pigment oxidation also stimulates lipid oxidation, which releases free radicals that oxidize iron and denature myoglobin molecules, causing meat color loss. Although several variables could impact the meat's color stability, the primary cause is myoglobin production due to free radicals. The C showed less yellowness (b*) during storage than the AMEUS. Increasing the AMEUS‐added concentration raises the b* value, whereas storing causes it to fall. Between 0 and 12 days of storage, the values for the AMEUS‐treated sample were 15.91, 15.23, 15.11, and 14.31, whereas for the C‐treated sample, they were 15.51, 13.57, 13.34, and 11.41, respectively. The carotenoids found in moringa leaves may be responsible for the processed pork patties' elevated b*. Although the dye's concentration dropped with time in storage, it rose sharply after AMEUS addition relative to C. Mashau et al. (2021) examined the effects of moringa leaf extract concentrations on minced beef and found similar results. After addition extraction, the treated samples had higher L*, a*, and b* values than the control sample. As storage time extended, all extract‐treated and control sample values declined.

Table 5 shows substantial (*p* < .05) effects of AMEUS addition at 0.5% and different storage periods on beef patties' pH levels. AMEUS‐treated patties had pH values of 6.36–7.11 after 12 days of storage compared to 5.68 for C‐treated patties. Gram‐negative bacteria like *Acinetobacter* and *Pseudomonas* flourish in high‐pH settings because they break down proteins and isolate amine groups (Das et al., 2011). The findings corroborated those of Khomola et al. (2021), who noted that the control sample's water‐carrying capacity decreased with time due to the addition of AMEUS; however, this loss was less severe than C.

**TABLE 5:** Pigments of patties treated by US and C during the storage period.

Pigments	Treatments	Storage period (day)			
		0	4	8	12
Myoglobin %	AMEUS	35.22 ± 0.95^a^	29.81 ± 0.82^c^	24.54 ± 0.81^d^	20.89 ± 0.84^f^
	C	34.81 ± 0.85^b^	29.12 ± 0.88^c^	22.35 ± 63^e^	18.12 ± 0.82^g^
Oxymyoglobin %	AMEUS	45.52 ± 0.93^A^	42.61 ± 0.57^B^	36.97 ± 0.60^D^	36.79 ± 0.71^DF^
	C	45.49 ± 0.93^A^	40.16 ± 0.73^C^	35.71 ± 0.78^E^	29.59 ± 0.79^G^
Metmyoglobin %	AMEUS	19.27 ± 0.65^a^	27.59 ± 0.75^b^	38.51 ± 0.75^d^	42.27 ± 0.98^e^
	C	19.31 ± 0.59^a^	30.91 ± 0.50^c^	42.21 ± 0.49^e^	53.41 ± 0.68^f^

*Note*: The different letters refer to significant differences among treatments at a level of .05.

Abbreviations: AMEUS, aqueous moringa extract by ultrasonic; C, control.

Table 4 displays the cooking characteristics, including the percentages of cooking yield and shrinkage due to cooking. “The results demonstrate a significant decrease (*p* ≤ .05) in the percentages of cooking yield for patties treated with AMEUS and C as the storage period increases. However, during the cold‐storage period of 12 days,” the decrease was more noticeable in the C treatment compared to the other treatments. The cooking yield in C decreased from 60.68% at the beginning of the storage period to 60.22% after 4 days, 59.95% after 8 days, and 59.11% at the end of the 12 days. In contrast, the decrease in AMEUS was less pronounced; it reached 70.57% at the beginning of the storage period, 67.11% after 4 days, and 64.52% after 8 days. The high concentration of active compounds and fibers in the extracts may be to blame; these components can hold water, which means that the cooking process results in less weight loss and a higher cooking output.

Regarding cooking‐induced shrinkage, the results demonstrate a statistically significant increase (*p* ≤ .05) in those percentages. However, the increase was more noticeable in (To) than in (T1); for example, during storage periods 0, 4, 8, and 12 days on, the percentage in (To) increased from 39.11% to 40.23%, then 41.52%, and finally 43.77%. In (T1), the rise was less pronounced, with results of 35.80%, 37.29%, 38.62%, and 39.36% for storage periods 0, 4, 8, and 12 days, respectively. Beef patties treated with the extract shrank in diameter at a slower rate than those untreated ones. This could be because the phenolic compounds in the extract improve the meat's capacity to retain water, which slows down the meat's weight loss while cooking.

The results were consistent with Khomola et al. (2021), who observed that beef patties with more moringa leaf powder cooked better than the control sample. Similar to the current study, Sharaf et al. (2009) observed that adding moringa leaf extract to preserved beef patties in various quantities boosted cooking yield percentages for the treated samples but decreased them for the control sample. Extract‐treated samples had somewhat higher cooking shrinkage percentages than controls.

### 3.11 Pigments

Table 5 results demonstrated that the impact of AMEUS addition and storage duration on the myoglobin pigment in cold‐stored beef patties was significantly different (*p* < .05). There was a discernible drop in pigment % in the control treatment (C), but no change in the AMEUS‐treated beef patties throughout the cold storage period. The proportion of myoglobin pigment in the C was 34.81% on the first day of storage, 29.12% after 4 days, 22.35% after 8 days, and a noticeable 18.12% after the 12 days storage period. The drop was less steep in the AMEUS, coming in at 35.22% before storage and 29.81% following 4 days. It reached 24.54% after 8 days and 20.89% after the 12 days storage period. Plant extracts are natural antioxidants with a solid capacity to reduce potential oxidation; this may explain why they did an excellent job preserving the natural pigments and why the extracts added to the meat kept the red color from fading (Karwowska & Dolatowski, 2007). In their investigation of the effects of plant extracts on meat pies, Baker et al. (2013) discovered that these extracts helped stabilize the color throughout storage, which is consistent with our results.

The addition of AMEUS considerably reduced the proportion of oxymyoglobin pigment in cold‐stored beef patties (*p* < .05) in Table 5, with the highest decrease in C, from 45.49% to 29.59% over 12 days. The AMEUS dye percentage peaked at 45.52% before storage, declined to 42.61% after 4 days, 36.97% after 8 days, and 36.79% after storage, showing a progressive reduction. This decline may be due to pigment oxidation into metmyoglobin. Mustafa (2013) found that pigment % reduces with storage time, while samples treated with green tea extract exhibited a reduced reduction than the control treatment, supporting that finding.

Table 5 shows a significant (*p* < .05) effect of AMEUS addition on the amount of methemoglobin pigment in beef patties. Aqueous AMEUS reduced methemoglobin pigment formation in beef patties compared to the control group. Over the course of storage time, the pigment percentage rose likewise. While the C‐treated beef patties rose more noticeably, the AMEUS‐treated ones rose less. It was 19.31% on the first day of storage and increased to 30.71% after 4 days. It rose to 42.21% after 8 days in storage. The treatment used as a control had 53.41% dye at the end of storage, indicating an increase. The proportion of dye before storage was 19.27% and grew to 42.27% after the 12 days storage period. However, this rise was reduced in beef patties treated with AMEUS.

Free radicals may have oxidized lipids, pigments, and metmyoglobin, explaining the increased pigment. The studied extract and other natural antioxidants prevent pigment production (Gallego et al., 2015). These findings supported Ibrahim et al. (2018), who examined how citrus peel extracts affected beef patties. They noted that the pies' color changed to brown while they were in storage due to metmyoglobin pigment synthesis, but that the AMEUS treatments had a lower concentration of brown color than the C. The findings were in line with those of Gallego et al. (2015), who used a vegetable extract in minced meat pies; they found that the pies treated with AMEUS had far better color than the C pies, even if there was a relative rise in methemoglobin pigment after storage. The red hue remained in the samples. This suggests that the plant extract can prevent or postpone the development of methemoglobin.

### 3.12 Sensory evaluation

**FIGURE 1:**
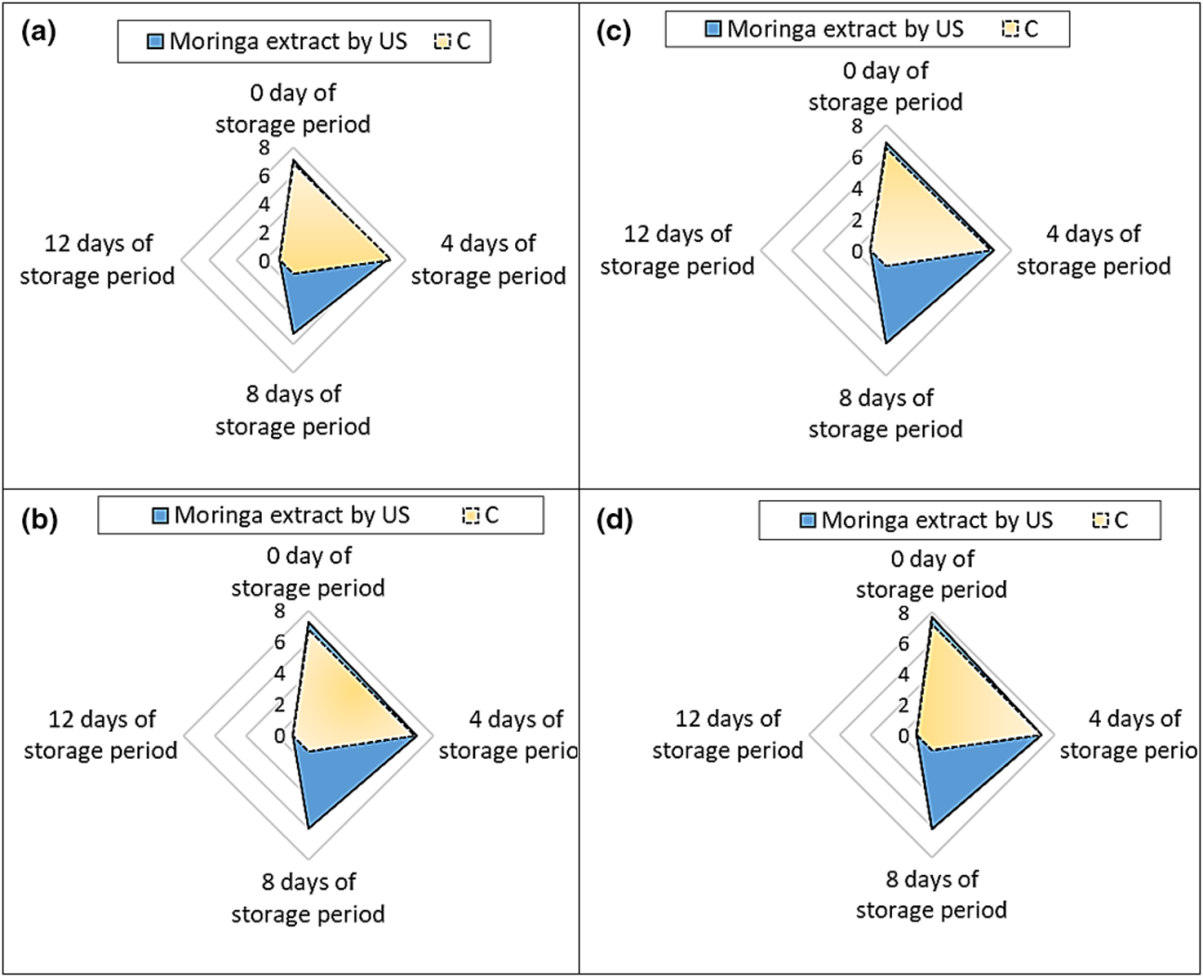
The sensory characteristics of the beef patties during the storage period. (a) Flavor, (b) tenderness, (c) juiciness, and (d) overall acceptability. C, control (without adding extract); US, ultrasound treatment.

Moringa stands out among the many plants abundant in vitamins, minerals, and necessary amino acids. In its role as a dietary supplement, it can help those who are undernourished due to illness or other causes. Figure 1 shows US and C moringa‐treated beef patties' flavor, softness, moisture, and acceptability from 0 to 12 days of storage. Findings showed that US‐made beef patties treated with moringa extracts had improved sensory characteristic ratings following 8 days of storage. The United States saw a decline from 7.15 to 5.23 for flavor, 7.22 to 5.97 for tenderness and juiciness, 6.87 to 5.95 for overall acceptability, and 7.23 to 6.11 for the overall acceptance of beef patties treated with moringa extracts. It took 8 days of storage for all sensory qualities to go down to 1 (poor). According to the results, beef patties treated with moringa extract had a shelf life of 4 days in instance C. These findings lead us to believe that American beef patties treated with moringa extract had a shelf life 100% longer than C. This was possible because the extracts contained bioactive chemicals, including flavonoids and phenolic compounds, which slowed the product's oxidation process and stopped the development of off‐putting smells and flavors. After 12 days, neither the US nor the C samples would consume moringa‐treated beef patties. The effects of chilling beef meatballs with different doses of moringa leaf extract for 18 days were similar to those of Abdallah et al. (2023). The researchers found that the flavor, softness, juiciness, and general acceptability somewhat improved, and the shelf life reached 9 days. They stated that the extract improved the product's sensory quality during storage. Rahman et al. (2020) found that moringa leaf extract improved the taste of goat meat patties. Evivie et al. (2015) discovered that moringa leaf extract lamb meatballs scored higher on sensory qualities.

## 4 CONCLUSION

Using ultrasonic technology to extract bioactive components from moringa has shown promise in enhancing antioxidant activity, increasing extraction yield, and developing a more efficient and ecofriendly extraction process. Processed beef patties exhibited beneficial antioxidant benefits when treated with AMEUS at a dosage of 0.5%. The processed patties may have reduced TBA and PV values because the extract, which contains antioxidant polyphenols, inhibits lipid oxidation. The extract increased protein and ash contents, decreased moisture and cooking loss, and increased cooking output and water‐holding capacity in the patties. Moringa leaf extract also kills food poisoning germs. Leaf extracts can improve meat products' shelf life and prevent foodborne viruses from spreading naturally, safely, and cheaply. Moringa leaf extract's polyphenols make it an excellent natural preservative for beef patties. Of the extract, 0.5% did not influence the patties' organoleptic properties.

## CONFLICT OF INTEREST STATEMENT

No conflict of interest.

## Supporting information


Data S1

## Data Availability

The data supporting the findings of this research are readily available in the manuscript.
