# Supplementary material for: Ultrasound‐assisted extraction of bioactive compounds from Moringa oleifera leaves for beef patties preservation: Antioxidant and inhibitory activities, half‐life, and sensory attributes
Source: Food Sci Nutr. 2024 Aug 9;12(10):7737–50. doi: 10.1002/fsn3.4395 (PMC11521678; doi:10.1002/fsn3.4395)
Supplement: Supplementary file 1 — Data S1 [file FSN3-12-7737-s001.docx]

**Table S1.** The chemical (%) and minerals (mg/100) composition of Moringa leaf powder

| chemical | | | | | | |
| --- | --- | --- | --- | --- | --- | --- |
| Carbohydrates | | Fiber | Ash | Fat | Protein | Moisture |
| 36.39±0.78 | | 19.27 ± | 6.88±0.72 | 5.11 ±0.80 | 27.37 ± 0.81 | 4.98 ±0.82 |
| minerals | | | | | | |
| Zn | K | Mg | Na | Fe | P | Ca |
| 3.22 | 1422.66 | 389.055 | 7.49 | 26.87 | 33.90.77 | 2031.55 |

**Table S2.** Polyphenols composition (ppm) from Moringa oleifera leaves

| Component | Formula | Reten. Time [min] | Area [%] | Concentration  (ppm) |
| --- | --- | --- | --- | --- |
| Qurcetine | C_15_H_10_O_7_ | 2.05 | 13.24 | 33.65 |
| Caffeic acid | C_9_H_8_O_4_ | 3.20 | 8.22 | 42.15 |
| Rutin | C_27_H_36_O_19_ | 4.19 | 11.65 | 62.15 |
| Kaempferol | C_15_H_10_O_6_ | 5.92 | 17.14 | 42.88 |
| Apigenin | C_15_H_10_O_5_ | 6.30 | 14.59 | 26.49 |
| Ferulic acid | C_10_H_10_O_4_ | 8.20 | 11.55 | 30.47 |
| Chlorogenic acid | C_16_H_18_O_9_ | 9.55 | 12.79 | 16.44 |
| gallic acid | C_7_H_6_O_5_ | 10.28 | 11.12 | 70.11 |

Table S3 Antibacterial activity of Moringa leaf extracts

| Diameter inhibition zones of bacteria test /mm | | | | |
| --- | --- | --- | --- | --- |
| Conc. % | *E. coli,* | *S. aureus* | *P. aeruginosa* | *B. cereus* |
| 0. 5 | 7±0.81 | 10±1.24 | 8±1.63 | 11±0.94 |

Table S4. First- and zero-order kinetic models rate constants, half-life, and statistics.

| Treatments |  | Zero order model | | | First order model | | | |
| --- | --- | --- | --- | --- | --- | --- | --- | --- |
|  | k0 | RMSE | R^2^ | t_1/2_ (day) | k1 | RMSE | R^2^ | t_1/2_(day) |
| US | 0.19610 | 0.086541 | 0.996202 | 3.53453 | 0.32974 | 0.64944 | 0.85440 | 2.10208 |
| C | 0.49546 | 0.644545 | 0.963161 | 1.39898 | 0.39949 | 2.12292 | 0.74378 | 1.73506 |

US: ultrasound treatment, and C: conventional. The different letters indicate significant changes at a significance level of 0.05.


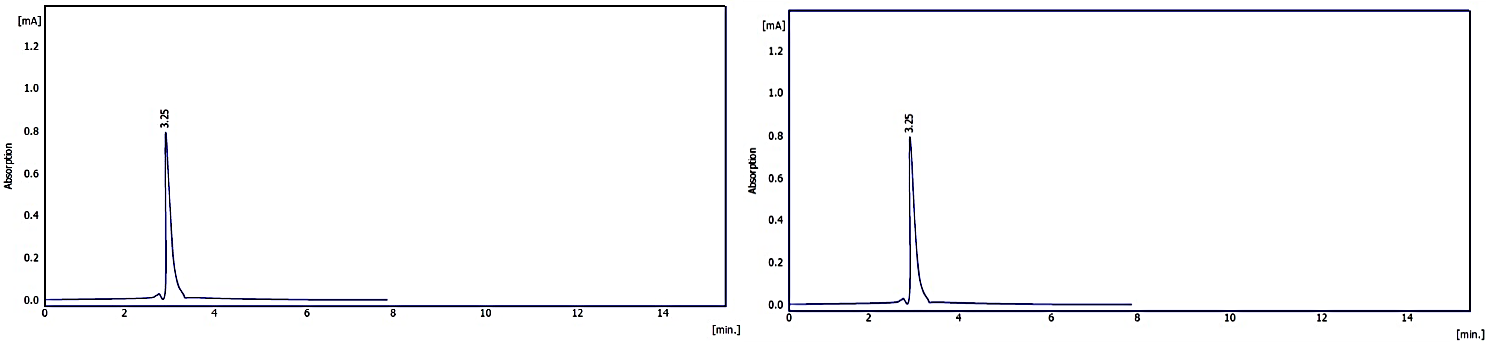


**B**

**A**


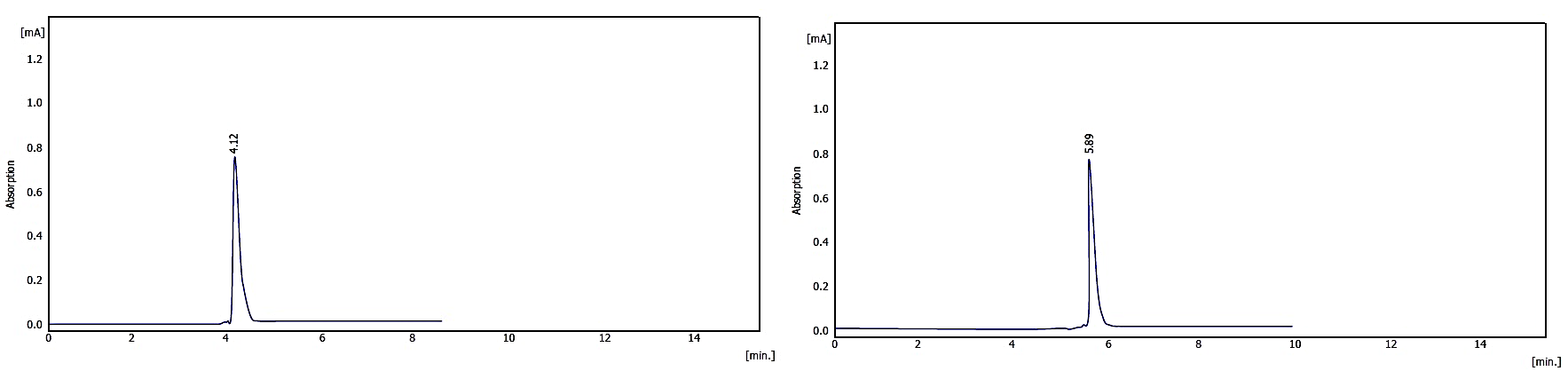


**D**

**C**


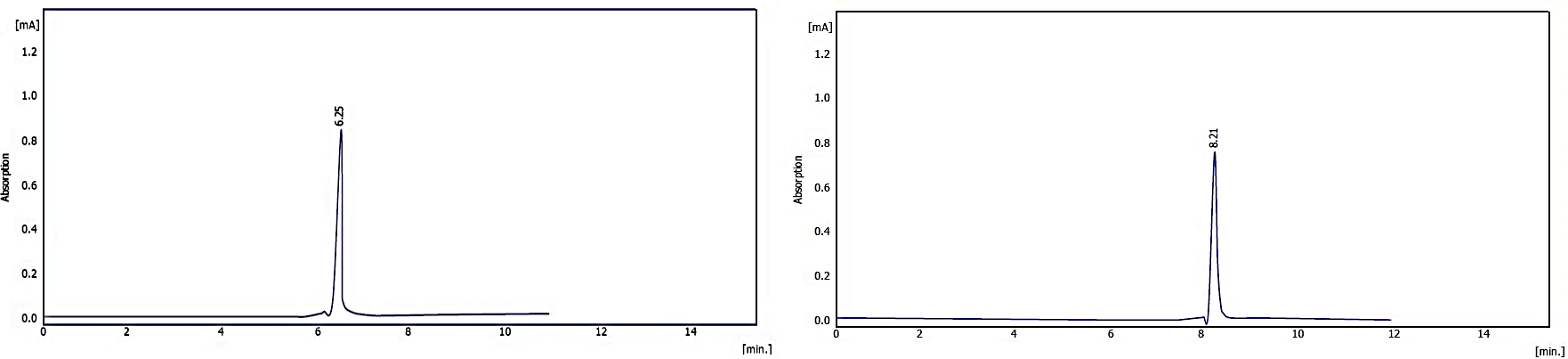


**E**

**F**


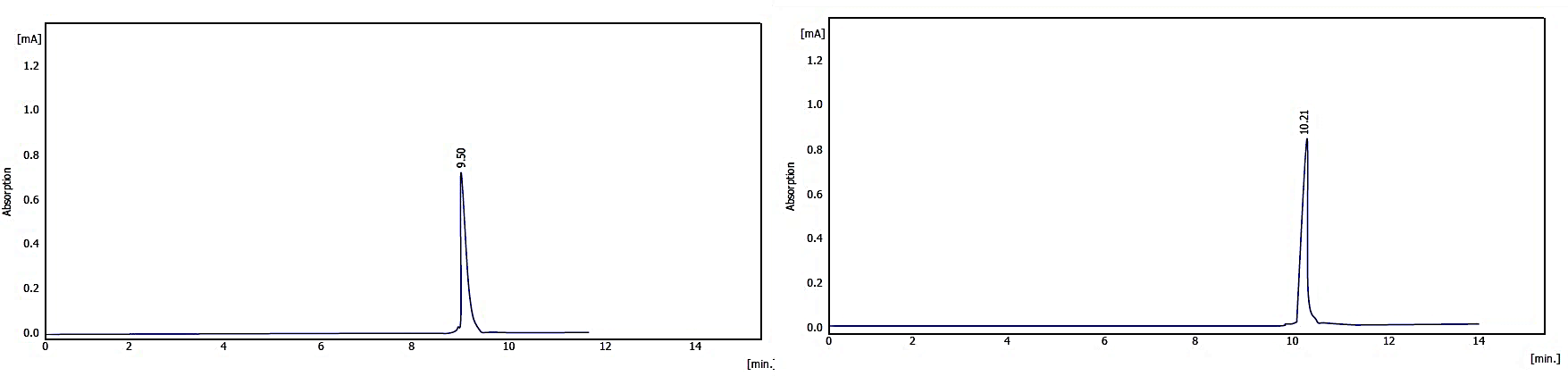


**H**

**G**

Figure S1. HPLC Chromatogram of standard compounds. A: Qurcetine, B: Caffeic acid, C: Rutin

D: Kaempferol, E: Apigenin, F: Ferulic acid, G: Chlorogenic acid, H: gallic acid

.


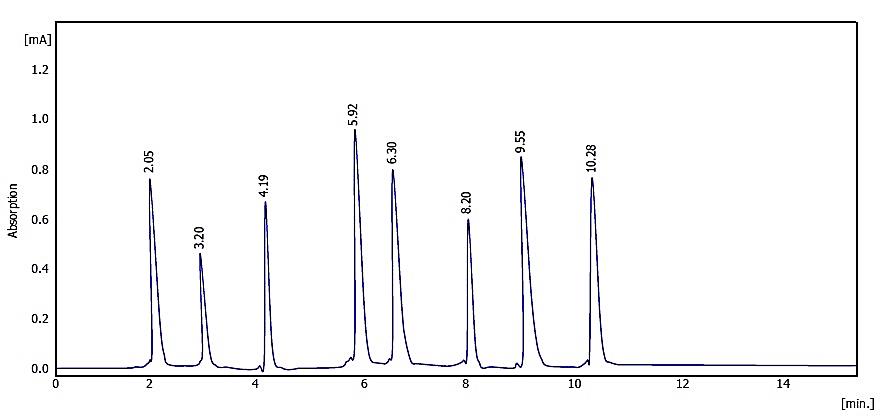


**Figure S2.** HPLC Chromatogram of *Moringa oleifera* leaves extracts
